# Crowdsourced Drug Discovery Approach Identifies BET Inhibitors As Novel Geroprotectors Promoting Healthy Aging

**DOI:** 10.1093/geroni/igaf122.3908

**Published:** 2025-12-31

**Authors:** Byungju Kim, Samuel Beck

**Affiliations:** Boston University, Boston, Massachusetts, United States; Boston University, Boston, Massachusetts, United States

## Abstract

The significant growth of the elderly population, driven by advances in biomedical science and public health, poses a critical socio-economic challenge. Identification and targeting of reliable biomarkers are essential for developing effective strategies to extend healthspan and lifespan. One promising target is age-associated misexpression of genes lacking CpG islands (CGI- genes), which is commonly observed across a broad range of aged tissues and contributes to chronic inflammation and loss of cell identity. Notably, established anti-aging interventions, such as calorie restriction, fasting, and a methionine-restriction diet, inhibits this dysregulation, suggesting that pharmacological suppression of CGI- gene misexpression would provide an effective strategy to promote healthy aging. To streamline this process, we developed a novel in silico drug discovery approach leveraging crowdsourced transcriptomic data and machine learning. Our approach significantly outperformed existing resources such as Connectivity Map, which exhibit crucial weaknesses in capturing pathological gene expression patterns in vivo. We analyzed CGI- gene expression in drug-treated vs. control groups using over 100,000 GEO samples and identified BET-inhibitors as potential geroprotectors suppressing CGI- gene misexpressions. Our experimental validation demonstrates that long-term (14-week) treatment with BET inhibitor effectively suppresses age-associated decline in muscle strength, body balance, and memory function, delays epigenetic aging, and promotes better survival in aged mice. Taken together, our study demonstrates that a pharmacological targeting of CGI- gene misexpression using a BET inhibitor provides an effective strategy for promoting healthy aging. In addition, our novel crowdsourced drug discovery approach offers an effective and practical solution for combating aging and associated diseases.

